# Tidyproteomics: an open-source R package and data object for quantitative proteomics post analysis and visualization

**DOI:** 10.1186/s12859-023-05360-7

**Published:** 2023-06-06

**Authors:** Jeff Jones, Elliot J. MacKrell, Ting-Yu Wang, Brett Lomenick, Michael L. Roukes, Tsui-Fen Chou

**Affiliations:** 1 Proteome Exploration Laboratory, Beckman Institute, California Institute of Technology, Pasadena, CA 91125 USA; 2 Division of Physics, Mathematics and Astronomy, California Institute of Technology, 1200 East California Boulevard, Pasadena, CA 91125 USA; 3 Division of Chemistry and Chemical Engineering, California Institute of Technology, 1200 East California Boulevard, Pasadena, CA 91125 USA; 4 Division of Biology and Biological Engineering, California Institute of Technology, Pasadena, CA 91125 USA

**Keywords:** Proteomics, Analysis, Quantitative, Pipeline, Workflow, Normalization, Imputation, Protein expression, Annotation enrichment

## Abstract

**Background:**

The analysis of mass spectrometry-based quantitative proteomics data can be challenging given the variety of established analysis platforms, the differences in reporting formats, and a general lack of approachable standardized post-processing analyses such as sample group statistics, quantitative variation and even data filtering. We developed *tidyproteomics* to facilitate basic analysis, improve data interoperability and potentially ease the integration of new processing algorithms, mainly through the use of a simplified data-object.

**Results:**

The R package *tidyproteomics* was developed as both a framework for standardizing quantitative proteomics data and a platform for analysis workflows, containing discrete functions that can be connected end-to-end, thus making it easier to define complex analyses by breaking them into small stepwise units. Additionally, as with any analysis workflow, choices made during analysis can have large impacts on the results and as such, *tidyproteomics* allows researchers to string each function together in any order, select from a variety of options and in some cases develop and incorporate custom algorithms.

**Conclusions:**

*Tidyproteomics* aims to simplify data exploration from multiple platforms, provide control over individual functions and analysis order, and serve as a tool to assemble complex repeatable processing workflows in a logical flow. Datasets in *tidyproteomics* are easy to work with, have a structure that allows for biological annotations to be added, and come with a framework for developing additional analysis tools. The consistent data structure and accessible analysis and plotting tools also offers a way for researchers to save time on mundane data manipulation tasks.

**Supplementary Information:**

The online version contains supplementary material available at 10.1186/s12859-023-05360-7.

## Background

**Fig. 1:**
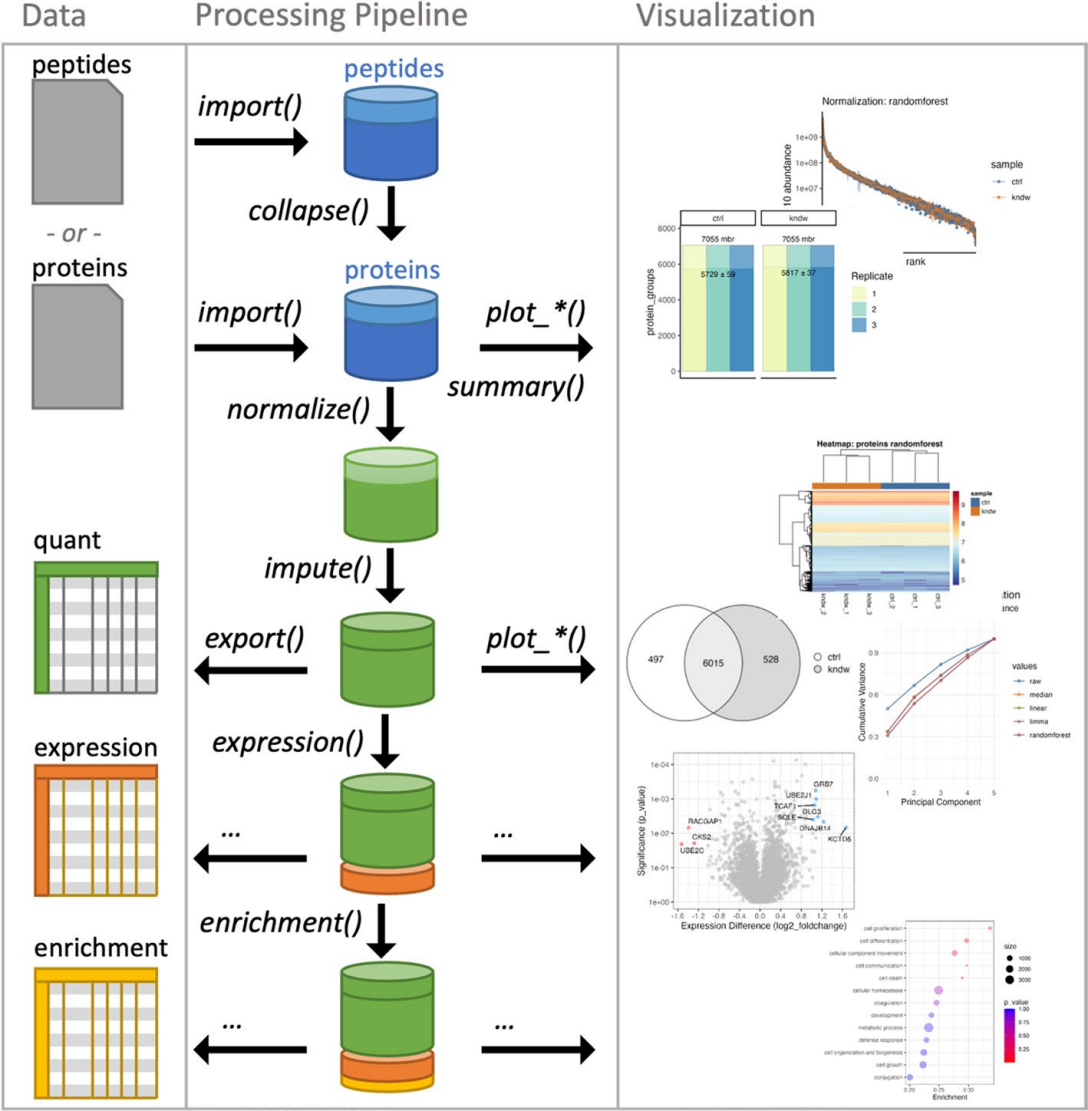
Shows a diagram of a typical workflow for quantitative protein analysis starting by importing quantitative peptide values from an external source, with each function responsible for transforming, analyzing and visualizing the data

Quantitative proteomics is at the forefront of translational biology [1, 2] and biomarker discovery [3–7], providing unparalleled access to the workings of complex biological systems. As such, there are numerous hardware and software platforms for measuring [8–11] and cataloguing quantitative proteomes [12–16], each with individualized methods of sorting, filtering, transforming quantitative values and visualizing the data [17]. Many of these tools generate output formats that have mixed data structures, non-standardized variable formats, and often confusing variable names. This can lead researchers to create one-off scripts for importing, cleaning, and analyzing data, often creating an environment of unmaintained code. Several R based packages already exist for the post analysis of quantitative proteomics data such as pmartR [18], protti [19] and DEqMS [20], along with several R packages that have companion web-based implementations such as MSstats [21, 22], DAPAR [23, 24] and ProteoSign [25]. In addition, there are a collection of software tools that implement a specific set of methods or lack accommodations to import data from multiple analysis platforms [17, 26–35]. We present *tidyproteomics,* an open-source R package for the post-analysis and visualization of proteomic data which aims to facilitate data explorations from multiple platforms, control over individual functions and analysis order, and serve as a tool to assemble complex processing workflows in a logical flow. This package takes inspiration from the tidyverse collection, which aims to share an “underlying design philosophy, grammar, and data structures” whereby data manipulation operations can be strung together end-to-end, or “pipelined,” using simple logical functions to transform and visualize data as diagrammed in Fig. 1. *tidyproteomics*, as an open-source package, aims to provide a platform for community standard analyses and visualizations that often require complex code to manipulate data differently for each analysis or visualization. For example, plotting a bar graph of protein counts per LCMS (liquid chromatography mass spectrometry) run might be straightforward in the R tidyverse packages, whereas plotting a Venn diagram of the protein count overlaps between samples requires inconsistent and advanced data manipulation. *tidyproteomics* attempts to bridge that gap for proteomics analysis by creating an intuitive and user-friendly environment for quantitative bioinformatics. Careful consideration was given to allow for full control over the order of operations, endowing users with the freedom to break convention. For example, the choice to normalize prior to imputation, or vice versa, has been explored and concluded [36], yet should remain a choice. Additionally, the choice to filter out contamination prior to normalization is advisable when it varies between samples, such as human keratin contamination [37]. However, for example, filtering out a deliberate co-cultured organism might be preferred post- normalization. Each of these “last-mile” analysis considerations, requires a simple and facile implementation for exploration, which is the ultimate goal for *tidyproteomics*. Generally, pre-processing operations such as *impute()*, *normalize()*, and *subset()* can be specified in a user-desired order, while post-processing operations for quality control, expression visualization, and ontology enrichment can be inserted at arbitrary steps along the pipeline.

### Implementation

This package is intended to serve varying degrees of R programming expertise, catering to novices with a companion web-based R Shiny app, enabling proficient R programmers to dictate nuanced control, and allowing experts to adapt, modify and extend the current codebase. This package contains numerous functions, each with variable parameters, and as such not all aspects are fully discussed herein, but in addition can be found in the online documentation. Furthermore, utilization of this package requires a basic understanding of R along with some cursory knowledge of LCMS based proteomics to properly employ. To help facilitate the utility of *tidyproteomics* among users without much R experience, a publicly available data set is pre-loaded at installation [38], and a web-based application has also been developed. Note that *tidyproteomics* is purely intended to facilitate the manipulation and analysis of LCMS based quantitative proteomics data (both labeled and label-free) and many of the analytical choices, such as normalization type or when to impute, are not proposed or enforced. It is therefore recommended to have a solid understanding in a suitable analytical approach, and many of the references cited herein provide a decent starting point. In depth guidance, discussion and examples can be found on the GitHub page.

### Extensibility

Flexibility for additional data processing platforms is supported through the use of import directives that define or translate the output data for consumption into tidyproteomics. This functionality is explained in detail in the R package accompanying documentation found on-line and should be able to accommodate any flat file schema.

Additionally, some functions allow for user-defined methods, such as calculating missing values with the *impute()* function, as well as the estimation of significance in differential expression between two groups using the *expression()* function. Although certain functions, such as *normalize()*, do not have plugins implemented, the framework should allow experienced R users to implement a desired method either by modifying the code on their own or submitting a request to the maintainers. The online documentation provides valuable resources and detailed explanations of the various features and functions available in the package, including how to implement custom methods and perform advanced data analysis. Analysis outside of this package is supported with the ability to convert the data-object to an R *data.frame* in either long or wide format extending the common *as.data.frame()*.

### Data importing

Importing data into *tidyproteomics* is handled by the main *import()* function, which currently can handle the output data from several common quantitative proteomics data processing suites such as ProteomeDiscoverer, MaxQuant, Skyline and DIA-NN. In addition to the native support for these platforms, there is a mechanism to create a configuration file to import data from almost any source. The data import process attempts to normalize the data structure into four basic components, each with simplified data structures as shown in Table 1. This function currently can accommodate either quantitative protein or peptide data, the latter of which can be converted to protein-level data via the *collapse()* function described later. In essence, this strategy conforms to the basic philosophy within modern data structures of storing redundant information in separate tables, reducing the size and complexity of a single table and thereby increasing the speed of accessing key components. As such, metadata pertaining to biological aspects such as GO, KEGG and UniProt annotations reside in a separate table called *annotations* and can be added without disturbing the main quantitative data while retaining utility in filtering and summary functions.

**Table 1:** The main data import structure utilizes a fragmented non-redundant scheme to minimize the size and complexity of the data

Data	Variable	Description
Experiments	sample_id	An 8-character string identifier
	import_file	The import file
	sample_file	The individual LCMS sample file
	sample	The sample name
	replicate	The sample replicate
Quantitative	sample_id	...
	sample	...
	replicate	...
	identifier	Proteins: protein Peptides: protein, peptide, modification
	abundance_…	the quantitative accounting value, existing as *raw* and "normalized" (eg. *median*, *linear*, *loess*, *randomforest*)
Accounting	sample_id	...
	identifier	Proteins: protein Peptides: protein, peptide, modification
	imputed	0-1 value, indicating the ratio of peptides imputed
	num_…	an integer accounting of *peptides*, *unique_peptides* and *proteins*
Annotations	identifier	Proteins: protein Peptides: protein, peptide, modification
	term	The annotation group(eg. molecular function)
	annotation	The annotation name (eg. metal ion binding)

Additionally, the Annotations table is setup in a one-to-many organization that does not enforce rigidity of term definitions across all measurement variables (eg. for each protein)

### Data curation

One of the more versatile aspects of this R package is the ability to extensively curate and filter the data. The function *subset()* allows data to be easily filtered with simple semantic expressions, similar to how the filter function in the tidyverse [39] package dplyr [40] operates. This package also introduces two new operators that work as a regular expression filter (%like%) which can be used in the semantic expression to subset data based on pattern matching in variable groups. For example, the expression *!description %like% ‘ribosome’* would keep all proteins with a description that does not include the word *‘ribosome’*. Additionally, together with the *merge()* and *reassign()* functions, data can be combined from multiple sources, assigned to specific sample groups and analyzed in a single collective. Alternatively, for example, data can be separated, normalized and imputed independently then recombined back into a single collective for analysis and visualization.

**Fig. 2:**
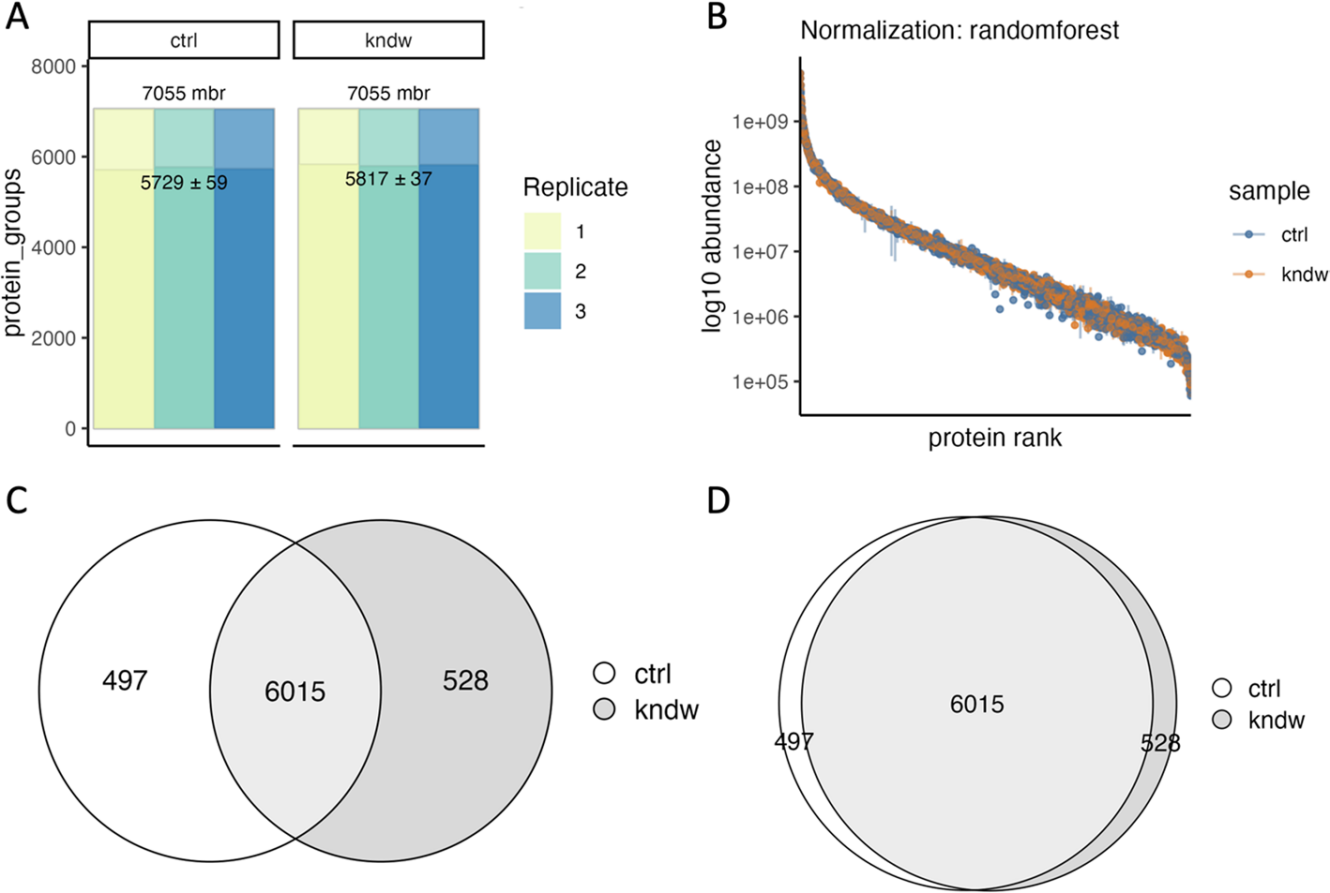
Summary statistic visualizations between control (ctrl) and knockdown (kndw) for **A** protein counts with match-between-runs (top) and without (bottom) with 95%CI shown, **B** the quantitative abundance for each protein rank ordered by abundance, and the without match-between-runs protein overlap **C** as a Venn diagram and **D** a Euler diagram

Once data are imported, the data object can immediately be summarized and visualized, showing the counts, quantitative dynamic range, and accounting overlaps (Fig. 2) to obtain a high-level perspective on the data. This includes the variation in measurement, which is important for understanding both the statistical power of the study and how it may improve through abundance imputation and normalization, as discussed in *Processing*. Additionally, all data can be exported to *csv, tsv* or other related tabular formats for analysis in other platforms.

### Annotations

Biological annotations are an important part of proteomic analyses as demonstrated by several methods that utilize conserved grouping nomenclature to derive multivariate insights [41–43]. The *tidyproteomics* package accommodates the incorporation of annotations from any source by linking the protein identifier with a term, such as Gene Ontology’s biological domains, and the associated annotation, such as catalytic activity or DNA repair. In essence, the annotation terms form the grouping structure for which all proteins belong to one or many of. This structure allows for several terms to be present simultaneously and used separately in enrichment analysis, including custom terms specific to a user’s research goals. Additionally, annotations can be harnessed in the *subset()* function and are additionally applicable in the *summary()* function, allowing users to quickly assess protein groups.

### Processing

Developing a quantitative proteomics data set requires some advanced knowledge of the experimental goals to determine whether steps such as normalization or imputation are warranted, and which specific methodologies should be employed. Several research articles have previously explored these topics [36, 44–50] and should be referenced in conjunction with specific analytical goals.

Table 2 attempts to summarize some of the more common proteomic experimental designs along with suggested analytical implementations.

**Table 2:** Suggested data normalization and imputation strategies for various proteomics experiments

Experimental design	Filter	Normalization	Imputation	Refs.
Small change between two groups (e.g. gene knockdown/out, mutation, disease, drug response, biomarker discovery)	n/a	*ANY*	Randomforest *BETWEEN*	[36, 58]
Difference between separate samples from the same organism (e.g. different organs, tissue sections, etc.)	n/a	Median shift	Randomforest *BETWEEN*	[36, 58]
Co-cultured multi-organism competitive study with or without environmental changes	*IN* single organism	Median shift	Randomforest *BETWEEN*	[59]
Affinity capture (flowthrough/capture)	*OUT* common contaminants	None, or linear based on bait subset	Minimum *WITHIN*	[60]
Antibody purification (flowthrough/capture)	*OUT* common contaminants	*n/a*	Minimum *WITHIN*	[60]
Protein over-expression	n/a	Median shift	Minimum *WITHIN*	[60]

These suggestions only reflect the opinions and experiences of the authors, have not been derived from examination of any specific literature, and do not come with any comparison testing. They are intended only as a starting point, adequate domain knowledge for each experimental design listed is expected

### Protein accounting

Central to proteomics is the need to assign peptides to proteins and accurately define differences in their quantitative abundances between conditions. *tidyproteomics* accommodates simple methods of protein accounting through the *collapse()* function, which takes in an imported quantified peptide data set and generates a protein data set according to several methods [51]. Unique to this function, however, is the ability to select the protein inferencing algorithm, the number of proteins, ranked by abundance, the use of a summary function (sum, median, mean, etc.) and the choice to split the abundance of shared peptides according to the summed proportion of each. However, it should be noted that more recent methods of protein accounting such as Tukey's Median Polish [35] and MaxLFQ [52] have not yet been integrated.

### Normalization

**Fig. 3:**
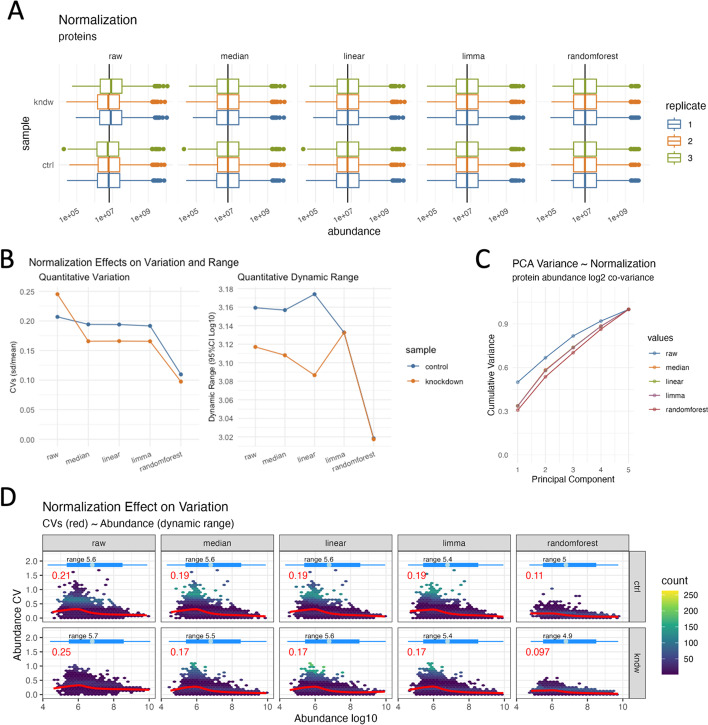
Post-normalization visualizations for **A** simple box plot of abundance (log10 scale) values for each normalization method, **B** effects of normalization on variance and dynamic range (95%CI Log10 abundance), note randomforest’s ability to dramatically lower the variance without effecting the overall dynamic range, which can also be visualized in **D** where the CVs (averaged red line, heat map dark blue hexagons) are plotted as a function of Log10 abundance, showing that higher CVs are prominent at lower abundances as expected, and **C** showing the cumulative variance from PCA analysis over the principal components

Quantitative proteomics relies on accurate normalization, for which several choices are available but remain somewhat difficult to accurately implement and may require distinct data formatting requirements. For example, a simple alignment of measured medians requires only a few lines of code, while implementing normalization from the *limma* package requires non-intuitive formatting of the data. The *normalize()* function is designed as a wrapper to handle various methods of normalization all at once, subsequently enabling researchers the ability to examine the result and choose the method best suited for their analysis. Alternatively, the *select_normalization()* function can automatically select the optimal normalization based on a weighted score combining coefficient of variation (CV), dynamic range (Fig. 3B) and variability in the first three principal component analysis (PCA) components (Fig. 3C) similar to other proposed methods [53], or the user can override this selection manually. The values from the selected normalization are then used for all downstream plots and analyses such as *expression()* and *enrichment()*. In addition to proteome-wide normalization, a subset can be used as the basis for normalization, such as for spike-in quantitative analytes or the bait protein in an immunoprecipitation experiment. This is accomplished with the same semantic syntax as with the *subset()* function and is reflected in the recorded operations.

### Imputation

**Fig. 4:**
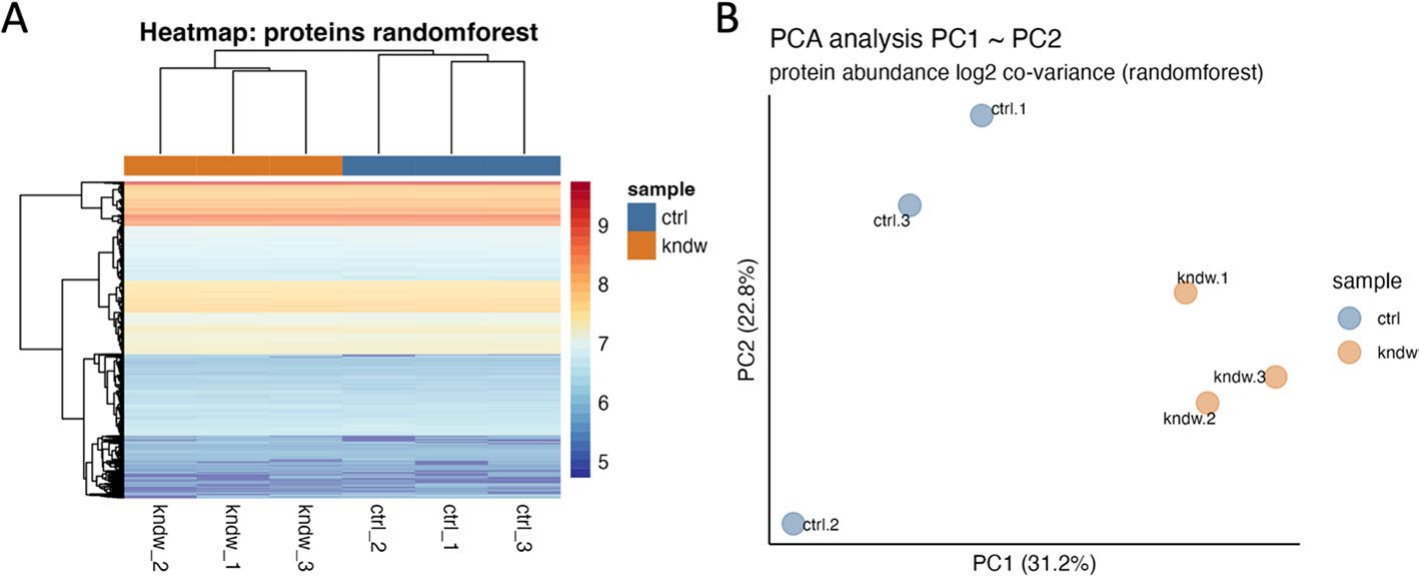
Post-normalization visualizations showing **A** hierarchal clustering heatmap and **B** PCA score plot of the first two principal components

Along with normalization, imputing missing values is another important task in quantitative proteomics that can be challenging to implement. Again, *tidyproteomics* attempts to facilitate this with the *impute()* function, which currently can support any base-level or user-defined function, applied either *within* or *between* sample groups. Additionally, the R package missForest [47] has been included and implemented to run in parallel to optimized computing times, which has been previously shown to yield the smallest error rates among algorithms evaluated for missing value imputation [36, 48–50]. Although random forest algorithms have demonstrated superiority in imputation and regression, this does not mean they should be used in every case. For example, when imputing missing values from a knock-out experiment, such as the one demonstrated herein and seen in Fig. 4, it can be preferrable to use *minimum* value imputation over the more complex *random forest*, simply because in this experiment we have an expectation that missing values are not at random, and likely due to our knockout procedure.

### Tracking

One of the more difficult challenges in data analysis is accurately and succinctly recording the chronological transformations that occur on a data set. *tidyproteomics* implements a simple system for tracking and recording each transformative step within the main data object. That record is also easily retrievable with the *operations()* function call, the result of which describes each transformation along with any relevant scientific literature citations.

### Visualization

The ability to summarize and visualize data, both pre and post processing, is critical to any processing pipeline. *Tidyproteomics* addresses this with both a *summary()* function and several *plot_()* functions. The summary function (described further in the online documentation) utilizes the same semantics inherent to *subset()* to generate summary statistics on any variable set, including all annotated and accounting terms. The functions *plot_counts()* and *plot_quantrank()* (Fig. 2 A and B, respectively) both utilize the summary function to provide summary statistic visualizations. Additionally, the *plot_venn()* and *plot_euler()* (Fig. 2 C and D, respectively) provide visualizations on the protein level overlap between 2 or more groups.

Visualizing processed data is an important aspect of data analysis, and great care is taken to explore the normalized data with a variety of plot functions (Fig. 3). Each of these is intended to display graphs that should lend insights such as the quantitative ranges pre and post-normalizations (Fig. 3A, *plot_normalization()*), the sample specific CVs and dynamic range (Fig. 3B, *plot_variation()*) and principal component variation (Fig. 3C, *plot_variation_pca()*) for each normalization. Perhaps more intriguing is the plot in Fig. 3D (*plot_dynamic_range()*) which shows a density heat map of sample specific CVs in relation to quantitative abundance. This plot highlights how CVs increase at the lower quantitative range and, more importantly, how each normalization method can address these large variances. Again, note how random forest normalization is best able to minimize the CVs at the lower quantitative range. Once normalization and imputation methods have been implemented and selected, it is often desired to visualize the unbiased clustering of samples. This can be accomplished with the *plot_heatmap()* and *plot_pca()* functions to generate plots as shown in Fig. 4 A and B, respectively.

## Results

The demonstration of the *tidyproteomics* functions are facilitated by data included within the package, which is the ProteomeDiscoverer 3.0 analysis of biological replicates of both a wildtype HCT116 cell culture (ctrl shRNA) and HCT116 cell culture with a single targeted knock-down (kndw, p97 shRNA) of the p97 gene [38]. The full analysis is provided in the Supplemental Materials as an example R script that can be used to generate all the figures shown here.

**Fig. 5:**
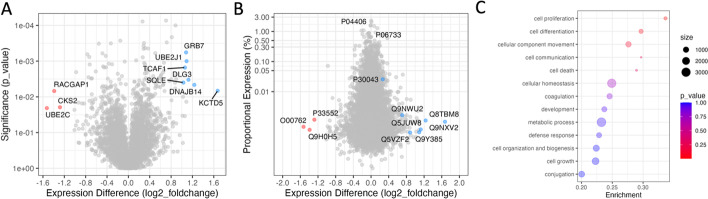
Differential expression analysis plotted as a **A** traditional volcano plot and as a **B** proportional plot that emphasizes the quantitative abundance of each protein, highlighting in color (red-downregulated and blue-upregulated) the proteins with statistical differences. Plot B is helpful when visualizing the results from a pull-down experiment where the differences are expected to be in the majority. Plot **C** visualizes the results from a term-enrichment analysis where terms are ranked by p_value

After data importing, filtering, normalization and imputation, a two-sample differential expression analysis can be initiated in *tidyproteomics* using the *expression()* function defined simply as the ratio of the two sample groups (e.g. *kndw/ctrl*) along with a chosen statistical method such as Student’s T-Test or an Empirical Bayes offered by the limma package[54], however, recent methods that consider PSM counts are not yet implemented [20, 29]. The resulting expression analysis can be visualized using the *plot_volcano()* and *plot_proportion()* plotting functions as shown in Fig. 5A and B. While the volcano plot depicted in Fig. 5A has long been the traditional visualization for expression data [55], the alternative plot in Fig. 5B has been influential in conveying the relative abundance of differentially expressed proteins when researchers are expecting their over-expression to have a dramatic effect, or are unaware of the overall proportion a targeted protein is within the dynamics of the entire proteome. In addition, we introduce a new visualization that compares the data between two expression analyses, which is accessible via *plot_compexp()*. This visualization is informative when comparing two different treatments against the same control (e.g. different compounds or separate gene mutations) and looking for similarities in significant protein expression differences (Additional file 1: Fig. S1). It can also be used to compare two different methods of determining expression differences in a single dataset, such as the Wilcoxon rank sum and Empirical Bayes methods (Additional file 1: Fig. S1). Furthermore, a term enrichment analysis is possible proceeding an expression analysis with the *enrichment()* function, again defined simply as the ratio of the two sample groups (e.g. *kndw/ctrl*) along with a chosen statistical method such as the gene-set enrichment analysis (GSEA) algorithm [41] or a simple Wilcoxon rank sum comparison. This analysis can be visualized with the *plot_enrichment()* function as shown in Fig. 5C.

## Conclusions

While there have been proposed data standards for quantitative mass spectrometry [56, 57], not all research conforms to the same formats. This R package addresses a small, but important, component of data interpolation between analysis platforms for efficient, simplified post-analysis of quantitative proteomic data. The datasets in *tidyproteomics* are easy to manipulate, model and visualize, and have a specific structure amenable to adding biological annotations for further analyses. The framework provided by *tidyproteomics* should also facilitate the development of additional tools for data analysis. The advantages of a consistent data structure and accessible analysis and plotting tools free researchers from mundane data manipulation tasks.

## Availability and requirements

Project name: tidyproteomics. Project homepage: https://github.com/jeffsocal/tidyproteomics. Operating system: platform independent. Programming language: R. Other requirements: none. License: MIT. Any restrictions to use by non-academics: none.

## Supplementary Information

**Additional file 1.** Supplemental provides an example R script utilizing the tidyproteomics package to demonstration the analysis pipeline and reproduce the figures used herein.

## Data Availability

The datasets analyzed within the current study are available in the *Tidyproteomics* code repository, https://github.com/jeffsocal/tidyproteomics and Shiny app https://github.com/ejmackrell/tidyproteomics-interactive. Access to both the protein and peptide data sets are immediately available upon loading the package. Additionally, the data set is available from the Caltech data repository, https://data.caltech.edu/records/aevwq-2ps50, taken from Wang et al. [38].

## Abbreviations

LCMS: Liquid chromatography mass spectrometry
PCA: Principal component analysis
CV: Coefficient of variation
GSEA: Gene-set enrichment analysis
