# Supplementary material for: Tidyproteomics: an open-source R package and data object for quantitative proteomics post analysis and visualization
Source: BMC Bioinformatics. 2023 Jun 6;24:239. doi: 10.1186/s12859-023-05360-7 (PMC10246047; doi:10.1186/s12859-023-05360-7)
Supplement: Supplementary file 1 — Additional file 1. Supplemental provides an example R script utilizing the tidyproteomics package to demonstration the analysis pipeline and reproduce the figures used herein. [file 12859_2023_5360_MOESM1_ESM.docx]

# Supplemental

An example R script utilizing the tidyproteomics package to demonstration the analysis pipeline and reproduce the figures used herein.

| 01 library(tidyverse)  02 library(tidyproteomics)  03  04 td <- path_to_package_data("p97KD_HCT116") %>%  05 # import the data set  06 import("ProteomeDiscoverer", "proteins") %>%  07 # change the sample labels  08 reassign("sample", "ctl", "ctrl") %>%  09 reassign("sample", "p97", "kndw") %>%  10 # save a table of simple summary stats  11 summary('sample', destination = 'save') %>%  12 # save a report on contamination  13 summary(contamination = 'CRAP', destination = 'save') %>%  14 # remove contamination  15 subset(!description %like% "^CRAP") %>%  16 # normalize via several methods, best method will be automatically selected  17 normalize(.method = c('median','linear','limma','randomforest')) %>%  18 # calculate the expression between experiment: ko and control: wt  19 expression(kndw/ctrl) %>%  20 # calculate the enrichment of the GO term(s) using the results  21 # from the expression analysis  22 enrichment(kndw/ctrl, .term = 'biological_process') %>%  23 enrichment(kndw/ctrl, .term = 'cellular_component') %>%  24 enrichment(kndw/ctrl, .term = 'molecular_function')  25  26 # save the data to this point  27 # downstream analysis may be tweaked for publication, no use re-computing  28 td %>% save_local()  29  30 # plot some simple summary stats  31 td %>%  32 # remove imputations for accounting stats  33 subset(match_between_runs == FALSE) %>%  34 subset(imputed == FALSE) %>%  35 plot_counts(destination = "png") %>% #FIGURE 2A  36 plot_quantrank(destination = "png") %>% #FIGURE 2B  37 plot_venn(destination = "png") %>% #FIGURE 2C  38 plot_euler(destination = "png") #FIGURE 2D  39  40 # plot visualizations comparing normalization methods  41 td <- td %>%  42 # remove imputations for accounting stats  43 subset(match_between_runs == FALSE) %>%  44 plot_normalization(destination = 'png') %>% #FIGURE 3A  45 plot_variation_cv(destination = 'png') %>% #FIGURE 3B  46 plot_variation_pca(destination = 'png') %>% #FIGURE 3C  47 plot_dynamic_range(destination = 'png') %>% #FIGURE 3D  48 # plot visualizations of unbiased clustering  49 plot_heatmap(destination = 'png') %>% #FIGURE 4A  50 plot_pca(destination = 'png') %>% #FIGURE 4B  51 # plot the expression analysis  52 plot_volcano(kndw/ctrl, destination = 'png',  53 significance_column = 'p_value') %>% #FIGURE 5A  54 plot_proportion(kndw/ctrl, destination = 'png') %>% #FIGURE 5B  55 # plot the enrichment analysis  56 plot_enrichment(kndw/ctrl, .term = 'biological_process',  57 destination = 'png') #FIGURE 5C  58  59 # SUPPLEMENTAL FIGURES  60 # run a an expression analysis using a t.test statistical comparison  61 tbl_expression_ttest <- rdata %>%  62 expression(kndw/ctrl, .method = stats::t.test) %>%  63 # export the results table to the assigned object  64 export_analysis(kndw/ctrl, .analysis = 'expression')  65  66 # run a an expression analysis using the limma statistical method  67 tbl_expression_limma <- rdata %>%  68 expression(kndw/ctrl, .method = 'limma') %>%  69 # export the results table to the assigned object  70 export_analysis(kndw/ctrl, .analysis = 'expression')  71  72 # plot the two expression tables two compare similarities between methods  73 plot_compexp(tbl_expression_ttest,  74 tbl_expression_limma,  75 labels_column = 'gene_name',  76 log2fc_min = 1, significance_column = 'p_value') +  77 ggplot2::labs(x = "(log2 FC) Wilcoxon Rank Sum",  78 y = "(log2 FC) Emperical Bayes (limma)")  )  80  81 ggsave("plot_enrichment_comparison.png",  82 width = 5, h = 4) #FIGURE S1 |
| --- |
|  |


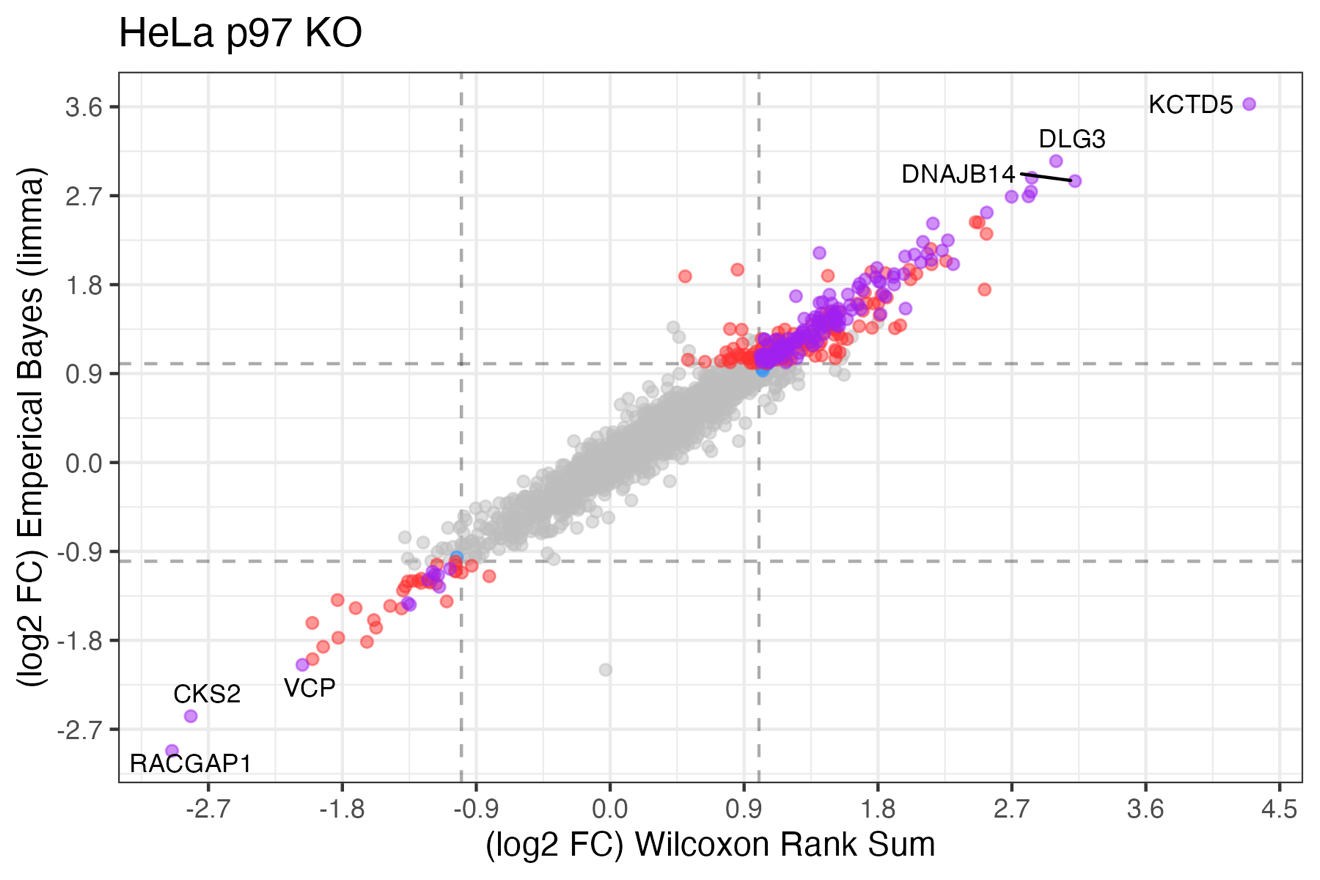


Supplemental Figure 1 demonstrates the comparison between two expression analyses. This plot is useful when two or more experimental conditions are present, such as compound A versus compound B with a control. The values are plotted as the Log2 fold-changes with the cutoffs for coloring the same as in a volcano plot, which are fold-change and significance thresholds. Here the significant proteins are colored red for only significant in abscissa (x-axis) values, blue for only significant in ordinate (y-axis) values, and purple having significance in both.
